# News and Events of the Week

**Published:** 1911-04-15

**Authors:** 


### NEWS AND EVENTS OF THE WEEK.

The Italian Government offers a prize of 10,000 lire for a work on anthrax infection prophylaxis. Papers, in Italian or French, must be sent to the Italian Minister of Agriculture before the first day of December 1911.

The fund for a Memorial to King Edward at Biarritz, where he went for six years, will be devoted to the Biarritz Nursing Home, which his late Majesty started, and to a Memorial in St. Andrew's English Church.

The eleventh medical study excursion will take place from August 28 to September 11, under the leadership of Prof. Landouzy, of Paris. The itinerary includes the south-eastern health-resorts of France. The secretary is Dr. C. de la Carriere, 2 rue Lincoln, Paris.

The King has granted to Dr. William Andrew Betts, of the Department of Public Health, Cairo, his Royal licence and authority to accept and wear the insignia of the Third Class of the Imperial Ottoman Order of the Medjidieh, which decoration has been conferred on him by the Khedive of Egypt, authorised by the Sultan of Turkey, in recognition of valuable services rendered by him.

